# Prognostic and predictive value of *PD-L2* DNA methylation and mRNA expression in melanoma

**DOI:** 10.1186/s13148-020-00883-9

**Published:** 2020-06-26

**Authors:** Friederike Hoffmann, Romina Zarbl, Dennis Niebel, Judith Sirokay, Anne Fröhlich, Christian Posch, Tobias A. W. Holderried, Peter Brossart, Gonzalo Saavedra, Pia Kuster, Sebastian Strieth, Gerrit H. Gielen, Sandra S. Ring, Jörn Dietrich, Torsten Pietsch, Lukas Flatz, Glen Kristiansen, Jennifer Landsberg, Dimo Dietrich

**Affiliations:** 1 Department of Dermatology and Allergology, University Hospital Bonn, Bonn, Germany; 2 Department of Otolaryngology, Head and Neck Surgery, University Hospital Bonn, Venusberg-Campus 1, 53127 Bonn, Germany; 3 Department of Dermatology and Allergology, Technical University of Munich, Munich, Germany; 4 Faculty of Medicine, Sigmund Freud University, Vienna, Austria; 5 Department of Oncology, Hematology and Rheumatology, University Hospital Bonn, Bonn, Germany; 6 Institute of Neuropathology, University Hospital Bonn, Bonn, Germany; 7 Microbiology and Immunology PhD Program, University of Zurich, Zurich, Switzerland; 8 Institute of Immunobiology, Kantonsspital St Gallen, St Gallen, Switzerland; 9 Department of Oncology and Hematology, Kantonsspital St Gallen, St Gallen, Switzerland; 10 Department of Dermatology, University Hospital Zurich, Zurich, Switzerland; 11 Department of Dermatology and Allergology, Kantonsspital St Gallen, St Gallen, Switzerland; 12 Institute of Pathology, University Hospital Bonn, Bonn, Germany

**Keywords:** *PD-L1*, *PD-L2*, DNA methylation, Melanoma, Prognostic biomarker, Predictive biomarker, Anti-PD-1 immunotherapy

## Abstract

**Background:**

PD-L1 (programmed cell death 1 ligand 1) expression in melanoma has been associated with a better response to anti-PD-1 (programmed cell death 1) therapy. However, patients with PD-L1-negative melanomas can respond to anti-PD-1 blockade, suggesting that the other PD-1 ligand, PD-L2 (programmed cell death 1 ligand 2), might also be relevant for efficacy of PD-1 inhibition. We investigated *PD-L2* expression and methylation as a prognostic and predictive biomarker in melanoma.

**Methods:**

DNA methylation at five CpG loci and gene expression of *PD-L2* were evaluated with regard to survival in 470 melanomas from The Cancer Genome Atlas. *PD-L2* promoter methylation in correlation with *PD-L2* mRNA and protein expression was analyzed in human melanoma cell lines. Prognostic and predictive value of *PD-L2* methylation was validated using quantitative methylation-specific PCR in a multicenter cohort of 129 melanoma patients receiving anti-PD-1 therapy. mRNA sequencing data of 121 melanoma patients receiving anti-PD-1 therapy provided by Liu et al. were analyzed for *PD-L2* mRNA expression.

**Results:**

We found significant correlations between *PD-L2* methylation and mRNA expression levels in melanoma tissues and cell lines. Interferon-γ inducible PD-L2 protein expression correlated with *PD-L2* promoter methylation in melanoma cells. *PD-L2* DNA promoter hypomethylation and high mRNA expression were found to be strong predictors of prolonged overall survival. In pre-treatment melanoma samples from patients receiving anti-PD-1 therapy, low *PD-L2* DNA methylation and high *PD-L2* mRNA expression predicted longer progression-free survival.

**Conclusion:**

PD-L2 expression seems to be regulated via DNA promoter methylation. *PD-L2* DNA methylation and mRNA expression may predict progression-free survival in melanoma patients receiving anti-PD-1 immunotherapy. Assessment of *PD-L2* should be included in further clinical trials with anti-PD-1 antibodies.

## Background

Immune checkpoint inhibitors of the programmed cell death (PD-1) pathway are able to induce dramatic and durable regression of metastatic melanoma, consequently leading to their regulatory approval in metastatic melanoma and also recently in the adjuvant setting [1, 2]. However, despite the tremendous success of immune checkpoint blockade, the majority of patients do not benefit with long-term remissions, and a relevant proportion of patients suffers from long-lasting immune-related side effects. Prognostic and predictive biomarkers are needed to identify patients at high risk of recurrence or progression and who are most likely to benefit from immunotherapy. To allow for the development of accurate predictive and prognostic biomarkers, knowledge on the regulation of immune checkpoint genes is mandatory.

PD-1 is a transmembrane receptor negatively regulating immune cells upon interaction with its two ligands PD-L1 (programmed cell death 1 ligand 1) or PD-L2 (programmed cell death 1 ligand 2). Receptor and ligands are key regulatory immune checkpoints that maintain self-tolerance by adjusting the degree of activation of immune cells [3]. Although the basal expression of PD-L2 seems to be lower compared to PD-L1, its affinity to the PD-1 receptor has been shown to be 2–6-fold higher than that of PD-L1 in human T cells [4]. PD-L2 expression can be induced by inflammatory cytokines on different immune and non-immune cells [5, 6]. Additionally, it can also be expressed by tumor cells including melanoma [7, 8]. PD-L1 protein expression measured by immunohistochemistry (IHC) has shown a positive correlation with response to anti-PD-1 blockade in multiple studies in a variety of tumor entities [9, 10]. However, patients with PD-L1 negative tumors can also benefit from anti-PD-1 blockade. Intratumoral heterogeneity of PD-L1 expression, the dynamic nature of PD-L1 expression in the tumor microenvironment, and the variability of detection methods can be explanations of the insufficiency of PD-L1 as a biomarker. Additionally, it has recently been shown that glycosylation of PD-L1 hinders its accurate immunohistochemical detection [11]. Other potential biomarkers, like density, phenotype, and diversity of tumor-infiltrating lymphocytes (TILs), tumor mutational burden, rare *JAK2* or *B2M* mutations, and specific gut microbial species can also correlate with response to anti-PD-1 therapy, but remain imperfect predictors of a response to PD-1 blockade [12]. The role for PD-L2 in predicting response to anti-PD-1 therapy has barely been investigated [9, 13]. Recently, in a cohort of pembrolizumab-treated patients with head and neck squamous cell carcinoma, PD-L2 positivity was significantly associated with response independent of PD-L1 status, and overall response rate was greatest in patients expressing both PD-L1 and PD-L2 ligands [8]. In patients with metastatic melanoma, PD-L1 and PD-L2 expression detected by IHC was associated with improved overall survival [7]. So far, the epigenetic regulation with particular focus on DNA promoter methylation of the PD-L2 encoding gene, *PDCD1LG2*, has not been considered as a biomarker in the context of anti-PD-1 immunotherapies in melanoma*.*

DNA methylation is an important epigenetic mechanism regulating the expression of proteins fundamental for T cell differentiation and T cell exhaustion [14–16]. Additionally, aberrant DNA methylation is an epigenetic hallmark of cancer and contributes to tumor progression by inactivating tumor suppressor genes [17]. It can function as a powerful biomarker that can reliably be detected and quantified even in limited amounts of formalin-fixed and paraffin-embedded (FFPE) tissues. A multitude of studies report on aberrant methylation of immune checkpoint genes, i.e. *PD-1*, *PD-L1*, and cytotoxic T-lymphocyte-associated protein 4 (*CTLA4*) in various malignancies [18–22]. In melanoma, *PD-L1* methylation regulates its expression and is associated with melanoma survival [23].

We recently reported on *CTLA4* DNA methylation as a potential biomarker predictive for immune checkpoint blockade efficacy [24]. In the present study, we identify methylated CpG (5'-cytosine-phosphate-guanosine-3) loci in the *PD-L2* promoter that correlate with mRNA expression in melanoma tissue and cell lines utilizing the The Cancer Genome Atlas (TCGA) cohort and 37 melanoma cell lines. Our survival analyses of our multicenter cohort of 129 melanoma samples prior to anti-PD-1 therapy and the TCGA cohort suggest that *PD-L2* DNA methylation might be a prognostic and predictive biomarker in melanoma. These findings are supplemented by recently published mRNA sequencing data of 121 melanoma patients prior to immune checkpoint blockade [25].

## Results

### Promoter methylation of *PD-L2* is inversely correlated with mRNA expression

**Fig. 1:**
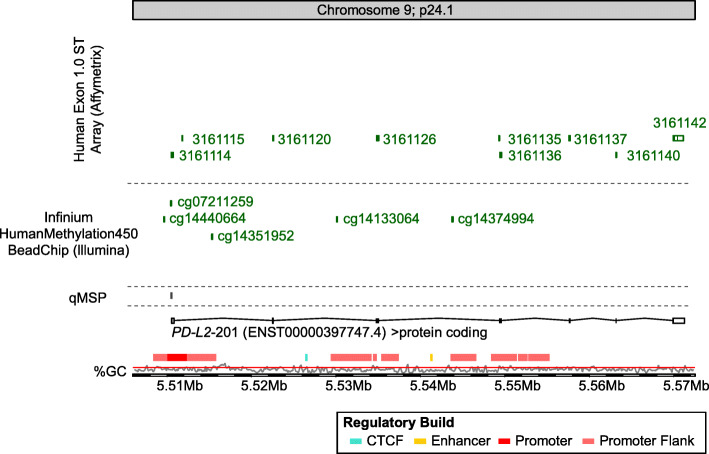
Genomic organization of the *PD-L2 (PDCD1LG2)* gene. Shown are regulatory elements, CG density, transcript variants, and target CpG sites of HumanMethylation450 BeadChip beads. The modified illustration was exported from https://www.ensemble.org (Release 95) and is based on Genome Reference Consortium Human Build 38 patch release 12 (GRCh38.p12)

The Infinium HumanMethylation450 BeadChip contains five beads targeting CpG sites within the *PD-L2* gene locus (Fig. 1). CpG site cg07211259 was located in the promoter region, cg14440664 and cg14351952 were situated in the promoter flanks, and cg14133064 and cg14374994 were located in the gene body.

We analyzed the correlation between methylation of the CpG sites and mRNA expression in *N* = 468 melanoma from the TCGA Research Network [26]. We found significant inverse correlations between *PD-L2* DNA methylation and mRNA expression levels at two out of five analyzed CpG sites (Table 1). Inverse correlations were strongest at cg07211259 located in the promoter region and cg14133064. Methylation of the other CpGs within the promoter flanks and the gene body showed a significant positive correlation with mRNA expression. These results suggest that PD-L2 and PD-L1 expression is regulated by gene methylation.

**Table 1:** Correlations of *PD-L2* methylation with mRNA expression, lymphocyte score, and overall survival

	Methylation or mRNA expression	Correlation with mRNA expression^†^		Correlation with lymphocyte score^†^		Overall survival (Cox proportional hazards)	
Analyte	Mean (95% confidence interval)	Spearman’s *ρ*	*P* value	Spearman’s *ρ*	*P* value	Hazard ratio (95% CI)	*P* value
*PD-L2* mRNA	94 (79–108)	NA	NA	**0.49**	**< 0.001**	0.85 (0.77–0.94)	**0.001**
cg14440664	72.0 (70.6–73.5)	**0.11**	**0.018**	0.10	0.078	0.63 (0.44–0.90)	0.012
cg07211259	25.6 (23.6–27.5)	**− 0.43**	**< 0.001**	− 0.22	**< 0.001**	1.16 (1.02–1.33)	0.027
cg14351952	80.0 (78.8–81.1)	**0.23**	**< 0.001**	− 0.02	0.78	0.58 (0.36–0.92)	**0.021**
cg14133064	51.8 (50.1–53.5)	**− 0.18**	**< 0.001**	**− 0.27**	**< 0.001**	0.84 (0.63–1.12)	0.24
cg14374994	86.6 (85.9–87.3)	**0.32**	**< 0.001**	0.05	0.40	0.30 (0.10–0.86)	**0.025**

*PD-L2* methylation was determined at five different CpG sites each gene targeted by HumanMethylation450 BeadChip beads (Fig. 1). *PD-L2* methylation and mRNA expression were analyzed as log2-transformed variable. Significant features are shown in boldface.

^†^*Correlations were performed including N = 468 (PD-L2 methylation and mRNA expression), N = 328 (lymphocyte score and PD-L2 mRNA expression), N = 329 (lymphocyte score and PD-L2 methylation) samples.*

### Association of *PD-L2* DNA methylation and mRNA expression with patients’ survival

**Fig. 2:**
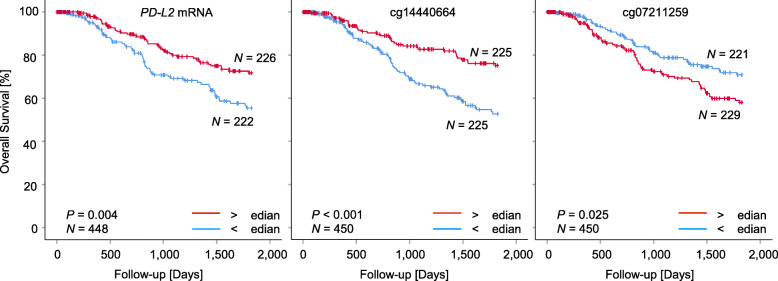
Overall survival in melanoma patients stratified according to *PD-L2* mRNA expression and methylation. Kaplan–Meier analysis of overall survival in melanoma patients stratified according to *PD-L2 *mRNA expression (left) and methylation levels of the indicated CpG sites. Only CpG sites showing significant survival differences are depicted. Patient samples were dichotomized based on median cutoffs. Follow-up data was available from *N* = 448 (mRNA) and *N* = 450 (methylation) patients

We investigated the relevance of *PD-L2* methylation and mRNA expression with patients’ overall survival. Methylation and mRNA expression levels were tested as continuous log2-transformed variates in order to avoid biases due to the introduction of cutoffs for patient sample classification. In univariate Cox proportional analysis, elevated *PD-L2* mRNA expression showed a significant correlation with better patients’ survival (Hazard ratio (HR)=0.85, 95% CI: 0.77–0.94; Table 1). A positive correlation between elevated methylation levels in the promoter flanks and the gene body (cg14440664, cg14351952, cg14374994) and better patients’ survival could be found. In contrast, elevated methylation levels at cg07211259 located in the promoter region were significantly correlated with poor outcome (Table 1). We further dichotomized mRNA levels and methylation levels based on optimized cutoffs for patient classification. Kaplan–Meier survival analyses confirmed better prognosis of patients with high *PD-L2* mRNA-expressing (above cutoff) tumors and tumors showing hypomethylation (below cutoff) at cg07211259 located in the promoter region (Fig. 2). In contrast to the CpG sites in the promoter region, hypermethylation of cg14440664 located in the promoter flank was associated with better patients’ overall survival.

### Promoter methylation of *PD-L2* is inversely correlated with immune cell infiltrates

**Fig. 3:**
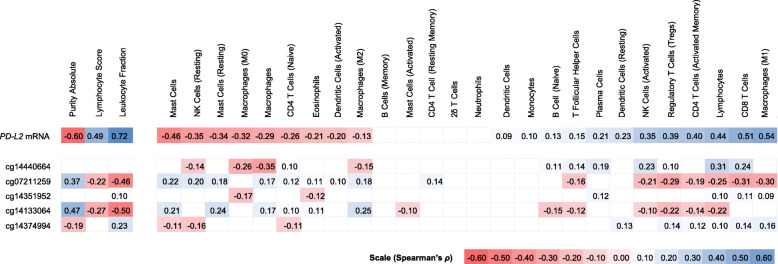
Heatmap of association between *PD-L2* CpG site methylation and mRNA expression with tumor-infiltrating lymphocytes (TILs) according to Thorsson et al. [27]. Shown are Spearman’s rank correlations (Spearman’s *ρ*) between methylation / mRNA expression of *PD-L2* and leukocyte fraction, as well as tumor-infiltrating leukocytes, including lymphocytes (CD8^+^ T cells, regulatory T cells, γδ T cells, naïve CD4^+^ T cells, resting and activated memory CD4^+^ T cells, naïve B cells, memory B cells, and resting and activated natural killer cells), monocytes and macrophages (M0 / M1 / M2 macrophages), resting and activated dendritic cells, resting and activated mast cells, eosinophils, and neutrophils. Immune signatures of tumor-infiltrating leukocytes were based on RNA-Seq analysis and the leukocyte fraction was based on methylation analysis. Only statistically significant (*P* < 0.05) are shown in color

Based on the assumption of a correlation between expression of *PD-L2* and adaptive immune cell activity, we tested the correlations between *PD-L2* mRNA levels and methylation with lymphocyte score and RNA-Seq signatures of TIL subsets as provided by Thorsson et al. [27]. We found a significant positive association between *PD-L2* mRNA expression and lymphocyte score. In accordance, we observed significant inverse associations between *PD-L2* methylation and lymphocyte score at cg07211259 and cg14133064 within the gene promoter and its flank (Table 1). Furthermore, in *PD-L2*, there were significant inverse correlations between cg07211259 and cg14133064 methylation and mRNA expression signatures of CD4^+^ T cells, regulatory T cells, activated NK cells, and lymphocytes (Fig. 3).

### Promoter methylation of *PD-L2* is inversely correlated with an interferon-γ signature

An interferon-γ (IFN-γ) signature has been described as a prognostic and predictive factor in melanoma [28, 29]. We therefore tested the correlations of *PD-L2* methylation and mRNA expression levels with an IFN-γ signature defined by the mRNA expression of *IFNG* itself and IFN-γ–regulated genes (*STAT1*, *STAT2*, *STAT3*, *JAK2*, and *IRF9*; Table 2). *PD-L2* mRNA expression levels were significantly correlated with an IFN-γ signature. Concordant inverse correlations between *PD-L2* methylation levels and IFN-γ signature were present predominantly in the promoter region at cg07211259. At cg14133064 in the promoter region, a significant inverse correlation between *PD-L2* methylation levels and *IFNG*, *STAT1*, and *IRF9* mRNA expression could be found. The remaining CpG sites showed positive correlations between methylation level and IFN-γ signature and did not significantly correlate with all IFN-γ–regulated genes.

**Table 2:** Correlations of *PD-L2* methylation and mRNA expression with IFN-γ signature

	*IFNG*		*STAT1*		*STAT2*		*STAT3*		*JAK2*		*IRF9*	
Analyte	Spearman’s *ρ*	*P* value	Spearman’s *ρ*	*P* value	Spearman’s *ρ*	*P* value	Spearman’s *ρ*	*P* value	Spearman’s *ρ*	*P* value	Spearman’s *ρ*	*P* value
*PD-L2* mRNA	**0.81**	**< 0.001**	**0.76**	**< 0.001**	0.32	**< 0.001**	**0.36**	**< 0.001**	**0.62**	**< 0.001**	**0.51**	**< 0.001**
cg14440664	**0.16**	**0.001**	**0.16**	**< 0.001**	**0.21**	**< 0.001**	0.04	0.45	**0.14**	**0.002**	**0.12**	**0.008**
cg07211259	**− 0.46**	**< 0.001**	**− 0.46**	**< 0.001**	**− 0.34**	**< 0.001**	**− 0.13**	**0.004**	**− 0.21**	**< 0.001**	**− 0.45**	**< 0.001**
cg14351952	**0.13**	**0.004**	**0.19**	**< 0.001**	**0.14**	**0.002**	0.08	0.086	**0.23**	**< 0.001**	**0.18**	**< 0.001**
cg14133064	**− 0.30**	**< 0.001**	**− 0.15**	**0.002**	0.03	0.57	**− 0.04**	0.35	**− 0.04**	0.42	**− 0.13**	**0.004**
cg14374994	**0.24**	**< 0.001**	**0.21**	**< 0.001**	0.06	0.23	**0.12**	**0.009**	**0.27**	**< 0.001**	**0.12**	**0.007**

Correlations of *PD-L2* methylation and mRNA expression with mRNA expression of *IFNG* and IFN-γ–regulated genes (*STAT1*, *STAT2*, *STAT3*, *JAK2*, and *IRF9*). DNA methylation was determined at five different CpG sites, each gene targeted by HumanMethylation450 BeadChip beads (Fig. 1). Significant features are shown in boldface. Data were procurable from *N* = 468 tumor samples

### *PD-L2* DNA methylation and mRNA expression in melanoma cell lines

PD-L2 can be expressed on melanoma cells as well as on tumor-infiltrating immune cells [30, 31]. Therefore, we wanted to test if the correlations between methylation and mRNA expression are simply a measure for the infiltration of PD-L2–expressing immune cells or if tumor cells themselves express PD-L2 epigenetically controlled. We analyzed the correlation between methylation of the CpG sites and mRNA expression in *N* = 37 melanoma cell lines [32]. In *PD-L2*, significant inverse correlation between DNA methylation and mRNA expression was found in the promoter region and its flank at cg07211259 and cg14351952 (Table 3).

**Table 3:** *PD-L2* methylation and mRNA expression in melanoma cell lines

	Methylation [%] or mRNA expression (mRNA: *N* = 19, methylation: *N* = 37)	Correlation with mRNA expression (*N* = 19)	
Analyte	Mean (95% confidence interval)	Spearman’s *ρ*	*P* value
*PD-L2* mRNA	6.9 (6.4–7.4)	NA	NA
cg14440664	58.1 (49.7–66.5)	− 0.379	0.11
cg07211259	26.4 (19.3–33.6)	**− 0.504**	**0.028**
cg14351952	68.5 (61.1–75.8)	**0.526**	**0.021**
cg14133064	53.0 (44.9–61.2)	− 0.016	0.96
cg14374994	86.2 (80.9–91.5)	0.333	0.16

*N* = 37 melanoma cell lines (*N* = 4 primary melanomas, *N* = 17 lymph node metastases, *N* = 16 brain metastases) obtained by Marzese et al. [32]. *PD-L2* methylation was determined at five different CpG sites, each gene targeted by HumanMethylation450 BeadChip beads (Fig. 1). Significant features are shown in boldface.

### Interferon-γ induces methylation-dependent PD-L2 protein expression

**Fig. 4:**
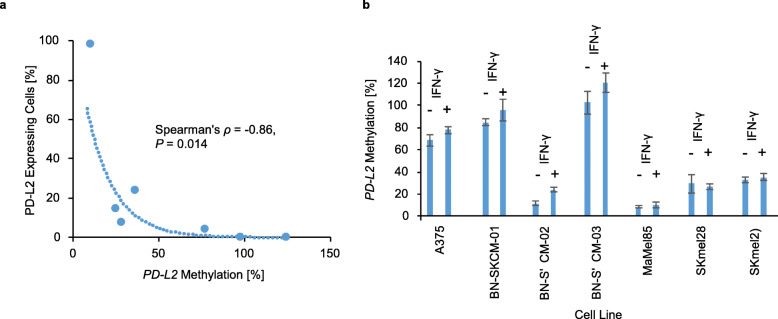
Impact of 72-h IFN-γ treatment on *PD-L2* methylation and PD-L2 protein expression in human melanoma cell lines*.* a *PD-L2* methylation in correlation to percentage of PD-L2 –expressing melanoma cells upon 72 h of IFN-γ treatment. b *PD-L2* methylation of the seven different human melanoma cell lines with or without interferon*-γ* treatment

In order to analyze the impact of IFN-γ on *PD-L2* DNA methylation and PD-L2 expression, we examined seven different human melanoma cell lines. Melanoma cell lines were treated with recombinant IFN-γ or left untreated for 72 h. Analyses were performed using a quantitative methylation-specific real-time PCR (qMSP) assay targeting CpG site cg07211259. PD-L2 expression was assessed via flow cytometry. 6/7 melanoma cell lines did not show a baseline PD-L2 expression. Only one cell line (SKmel29) displayed 3% of PD-L2–expressing melanoma cells. Therefore, no correlation could be found for *PD-L2* DNA methylation and PD-L2 expression in the absence of IFN-γ (*ρ* = − 0.43, *P* = 0.34). IFN-γ treatment for 72 h was able to induce PD-L2 expression in 5/7 melanoma cell lines. The two cell lines (BN-SKCM-01 and BN-SKCM-03) that did not respond to IFN-γ treatment with PD-L2 upregulation showed a higher *PD-L2* promotor methylation than IFN-γ responsive melanoma cell lines. IFN-γ–induced PD-L2 protein expression was significantly correlated with *PD-L2* methylation (*ρ* = − 0.86, *P* = 0.014; Fig. 4a). *PD-L2* methylation levels did not significantly alter upon 72 h of IFN-γ treatment (Fig. 4b).

### Prognostic and predictive value of *PD-L2* DNA methylation and mRNA expression in melanoma samples prior to anti-PD-1 therapy

**Fig. 5:**
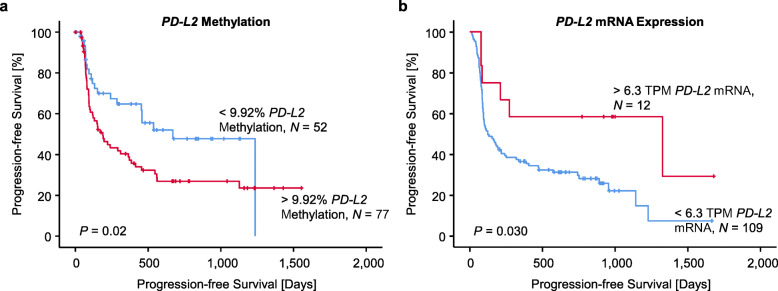
Progression-free survival in two cohorts of metastasized melanoma patients treated with immune checkpoint blockade in association to *PD-L2* methylation and *PD-L2* mRNA expression. Kaplan–Meier analysis of progression-free survival in metastasized melanoma patients stratified according to cg07211259 methylation (**a**) and *PD-L2* mRNA expression (b) levels in pre-treatment samples. Patient samples were dichotomized based on optimized cutoffs. Follow-up data was available from *N* = 129 (methylation cohort) and *N* = 121 (mRNA expression cohort obtained from Liu et al. [25]) patients, respectively

To investigate the prognostic and predictive value of *PD-L2* DNA methylation, we examined *PD-L2* DNA methylation in *N* = 129 patients with metastatic melanoma prior to anti-PD-1 immunotherapy. Patient characteristics are shown in Table 4. Analyses were performed using a qMSP assay targeting CpG site cg07211259. We found that *PD-L2* DNA methylation dichotomized based on an optimized cutoff (9.92%) was significantly correlated with patients’ progression-free survival under anti-PD-1 immunotherapy (*P* = 0.023; Fig. 5a). Patients with low methylation levels showed significant longer progression-free survival than patients with high methylation levels. We further analyzed RNA-Seq (data provided by Liu et al. [25]) of 121 melanoma patients receiving anti-PD-1 therapy. In accordance with our DNA methylation results, *PD-L2* mRNA expression dichotomized based on an optimized cutoff (6.3 Transcripts Per Million (TPM)) was correlated with patients’ progression-free survival (*P* = 0.030, Fig. 5b).

**Table 4:** Patient characteristics of the ICB cohort

Characteristics	Number *N*	(%)
All patients	129	100
Age in years, (range)	67.11, (28−92)	
Gender		
Female	50	38.8
Male	79	61.2
Site of melanoma metastases		
Cutaneous metastases	62	48.1
Lymph node metastases	37	28.7
Lung metastases	13	10.1
Brain metastases	11	8.5
Abdominal metastases	6	4.7
*BRAF* mutation status		
*BRAF* wild type	84	65.1
*BRAF* mutated	42	32.6
Unknown	3	2.3
Response to anti-PD-1 blockade		
Progressive disease (PD)	58	45.0
Partial response (PR)	39	30.2
Stable disease (SD)	8	6.2
Complete response (CR)	22	17.0
Unknown	2	1.6
Therapeutic regimen		
Anti-PD-1 monotherapy	68	52.7
Ipilimumab, anti-PD-1 monotherapy	12	9.3
Ipilimumab + nivolumab	17	13.2
Ipilimumab, ipilimumab + nivolumab	2	1.5
Anti-PD-1 monotherapy, ipilimumab + nivolumab	30	23.3
Medical center		
University Hospital Bonn—dermatology	104	80.6
University Hospital Bonn—oncology	5	3.9
University Hospital Bonn—neurosurgery/-oncology	4	3.1
Kantonsspital St. Gallen, Spital Grabs, Spital Wil, Spital Flawil	16	12.4

Data include age, gender, site of the melanoma metastases, *BRAF* mutation status, response to anti-PD-1 blockade, therapeutic regimen, and the treating medical center of *N* = 129 stage IV melanoma patients receiving immune checkpoint blockade

## Discussion

We found significant inverse correlations between *PD-L2* methylation and mRNA expression levels at all analyzed promoter CpG sites in melanoma tissue. Thus, our data indicate a gene silencing mechanism via promoter methylation. Significant inverse correlations between *PD-L2* methylation and mRNA expression levels at cg07211259 in the promoter region of melanoma cell lines confirmed that melanoma cells themselves express PD-L2 epigenetically controlled. A possible epigenetic regulation of the PD-1 ligands PD-L2 and PD-L1 via DNA methylation has already been postulated for other solid tumors [20, 33, 34]. In melanoma, regulation of PD-L1 expression by DNA promoter methylation has recently been demonstrated in three melanoma cell lines that showed increased PD-L1 expression upon treatment with the hypomethylating agent 5-azacytidine [23]. In our study, we identified significant inverse correlations between *PD-L2* DNA methylation and mRNA expression levels in melanoma, that were most pronounced at cg07211259 and cg14133064. CpG site cg07211259 is located in an upstream *PD-L2* promoter region. However, according to literature, a second downstream promoter might exist that functions in a lineage-specific fashion and regulates PD-L2 expression in B cells. This downstream promoter appears to be located between exon 1 and exon 2 of the main transcript variant [35]. This may explain the significant inverse correlation between DNA methylation and mRNA expression levels at cg14133064 in the TCGA cohort. Cell type-specific usage of alternative promoters in human genomes has already been described in literature [36]. We assume that this second downstream promoter might only be relevant in B cells, but not in melanoma cells. This would explain the insignificant correlation between cg14133064 methylation level and mRNA expression levels in melanoma cell lines.

A positive correlation between *PD-L2* mRNA expression and *PD-L2* DNA methylation could be detected at the remaining CpG sites located in the promoter flanks. Such extensive positive correlations between methylation and transcriptional activity are frequently found in gene bodies [37]. In conclusion, our data suggest that in accordance to *PD-L1*, *PD-L2* mRNA expression is regulated by promoter methylation in melanoma.

Further experiments are needed to establish a causal relationship between DNA methylation of the promoter region and PD-L2 transcription. In our study, we refrained from using demethylating agents as for example 5-aza-2-deoxycytidine, as it does not specifically demethylate *PD-L2*, but instead leads to an unspecific demethylation of the whole genome which may also include transcription factors and cytokines. A more specific and elegant approach could be a specific zinc finger-induced methylation of the *PD-L2* promoter, as it has been described by Li et al. in the context of *PD-L1* [38]. However, until now, this is not an established method.

The prognostic value of PD-L2/PD-L1 expression in melanoma is still unclear. PD-L1 protein expression of melanoma cells detected by IHC has been described as an independent prognostic factor for melanoma [39, 40]. Obeid et al. demonstrated that in 147 metastatic melanomas, PD-L1 and PD-L2 expression defined by positive IHC on tumor cells is associated with improved overall survival [7]. However, PD-L1 expression has also been reported to be correlated with an unfavorable prognosis in various malignancies, including melanoma [41]. In our study, *PD-L2* DNA promoter hypomethylation and high mRNA expression were found to be a predictor of prolonged overall survival in the absence of anti-PD-1 immunotherapy. In line with these results, we found *PD-L2* DNA methylation and mRNA expression to be correlated with known prognostic factors, such as tumor-infiltrating lymphocytes and an IFN-γ signature. Tumor-infiltrating lymphocytes are known to be associated with favorable prognosis in primary and advanced melanoma [42, 43]. An IFN-γ signature has been shown to be associated with response to immune checkpoint inhibitors [44, 45]. Thus, *PD-L2* DNA promoter hypomethylation and high mRNA expression might be prognostic factors, associated with improved overall survival in melanoma patients.

In order to further analyze the correlation between *PD-L2* DNA methylation and PD-L2 expression, we examined PD-L2 expression in human melanoma cell lines with and without IFN-γ treatment. As expected, treatment with IFN-γ did not change DNA methylation levels, but did increase PD-L2 expression. Hypermethylated melanoma cell lines showed a significantly lower PD-L2 expression after IFN-γ treatment than hypomethylated cell lines. This supports our hypothesis, that IFN-γ–induced PD-L2 expression in melanoma cells is controlled by the degree of DNA methylation of the *PD-L2* promoter region. Of note, we did not find a correlation between PD-L2 protein expression and *PD-L2* promoter methylation in the absence of IFN-γ. This fact might point towards the utility of *PD-L2* methylation testing to determine not only the effective, potentially transient PD-L2 protein expression but also the general ability of melanoma cells to express PD-L2 protein under specific proinflammatory conditions. This finding would suggest a higher biomarker performance of *PD-L2* methylation compared to PD-L2 expression.

To increase therapeutic efficacy and reduce treatment-related morbidity, predictive and prognostic biomarkers are urgently needed to identify patients that are most likely to benefit from checkpoint blockade. PD-L1 protein expression measured by IHC has been shown to be associated with response to anti-PD-1 therapy in certain studies, but its performance as predictive biomarker remains insufficient [12]. Our data suggest that *PD-L2* DNA promoter hypomethylation might be correlated with patients’ prolonged progression-free survival under anti-PD-1 antibody therapy. Consistent with our results obtained from the DNA methylation analysis, high *PD-L2* mRNA expression appears to be correlated with patients’ prolonged progression-free survival in metastatic melanoma patients under anti-PD-1 therapy provided by Liu et al. [25]. Thus, *PD-L2* DNA methylation and mRNA expression seem to be predictive biomarkers in melanoma patients receiving anti-PD-1 therapy.

Our study covers only a limited number of CpG sites within *PD-L2*. Hence, further studies, i.e., using bisulfite sequencing, should be performed to identify the CpG sites showing the highest biomarker performance. A special focus should be placed on CpG sites in enhancers and potential alternative promoters. As our analyzed CpGs sites already show a correlation of DNA methylation levels with mRNA expression and overall survival of melanoma patients, it is possible that other CpG sites will exhibit an even better biomarker performance.

We are aware of the limitations of our present study. The analysis of multiple CpG sites is, in general, vulnerable to multiple testing issues. Results would therefore have to be validated in an independent cohort. In order to reduce the risk of multiple testing artifacts, we reported the unselected results for all CpG sites analyzed in our study. Furthermore, in our patient cohort receiving anti-PD-1 therapy, data were dichotomized based on optimized cutoffs. When dichotomized based on median cutoffs, significant results could not be found, which might be due to the small sample size of only 129 or 121 melanoma patients, respectively. Therefore, we cannot refer *PD-L2* as a definite predictive biomarker and further validation is required.

## Conclusion

Biomarkers that allow for the prediction of clinical response to anti-PD-1 therapy are desperately needed. Our data suggest an epigenetic regulation of PD-L2 expression via DNA methylation and a predictive value for progression-free survival in anti-PD-1 treated melanoma patients. We conclude that in contrast to PD-L2 protein expression, *PD-L2* methylation allows to determine not only the effective expression status of melanoma cells, but also the ability to express PD-L2 under proinflammatory conditions, i.e., in the presence of IFN-γ. Assessment of *PD-L2* promoter methylation and expression testing therefore should be included in further clinical trials with anti-PD-1 antibodies.

## Material and methods

### Patients

#### TCGA cohort

The data we analyzed are partly based on datasets from The Cancer Genome Atlas Research Network (TCGA, http://cancergenome.nih.gov/). We included *N* = 470 primary solid and metastatic melanoma tumor tissue samples from the TCGA skin cutaneous melanoma (SKCM) cohort. The TCGA Research Network obtained informed consent from all patients in accordance with the Helsinki Declaration of 1975. One sample per patient was analyzed. In patients providing more than one sample, metastatic tumor samples were included. We used the TCGA Research Network to obtain supplementary clinico-pathological data. Molecular data were adopted from a study previously published by the TCGA Research Network [26]. Datasets comprising information about sample purity and ploidy estimates were adopted from the TCGA Research Network and calculated using the ABSOLUTE algorithm [46]. We used the results provided by Thorsson at al [27]. who developed RNA sequencing (RNA-Seq) signatures as a surrogate for immune cell infiltrates to obtain quantitative data on infiltrating leukocytes. Data on infiltrating lymphocytes were adopted from the TCGA Research Network [26], including lymphocyte distribution (0–3; 0 = no lymphocytes within the tissue, 1 = lymphocytes present involving < 25% of the tissue cross-sectional area, 2 = lymphocytes present in 25 to 50% of the tissue, 3 = lymphocytes present in > 50% of tissue), lymphocyte density (0–3; 0 = absent, 1 = mild, 2 = moderate, 3 = severe), and lymphocyte score (0–6, score defined as the sum of the lymphocyte distribution and density scores).

#### ICB melanoma cohort

Patients (*N* = 129) diagnosed with metastatic melanoma and treated with anti-PD-1 checkpoint inhibition at the University Hospital Bonn, the Kantonsspital St. Gallen, the Spital Grabs, the Spital Wil, and Spital Flawil between May 2012 and June 2019 were included in the cohort (patients characteristics are shown in Table 4). Response patterns were reported based on Response Evaluation Criteria in Solid Tumors (RECIST). The study protocol was approved by the Institutional Review Board.

#### Patient cohort provided by Liu et al. [25]

Advanced melanoma patients (*N* = 121) treated with an anti-PD-1 antibody alone or in combination with an anti-CLTA-4 antibody in a palliative setting were included in the cohort. Response patterns were reported based on RECIST criteria.

### Melanoma cell lines

We included *N* = 37 melanoma cell lines (*N* = 4 primary melanomas, *N* = 17 lymph node metastases, *N* = 16 brain metastases) obtained by Marzese et al. [32] (Gene Expression Omnibus (GEO) accession number: GSE44662; National Center for Biotechnology Information (NCBI), Bethesda, MD, USA). Furthermore, we included seven human melanoma cell lines (BN-SKCM-01, BN-SKCM-02, BN-SKCM-03, A375, MaMel85, SKmel28, SKmel29). The MaMel85 human melanoma cell line were established, characterized and kindly provided by Dirk Schadendorf (University Hospital Essen, Essen, Germany). BN-SKCM-01, BN-SKCM-02, and BN-SKCM-03 were originally isolated from melanoma metastases collected from adult donors treated at the University Hospital Bonn with participants’ informed consent. All human cells were cultured in complete RPMI 1640 medium supplemented with 10% FCS (Merck KGaA, Darmstadt, Germany), 2 mM L-glutamine (Life Technologies, Carlsbad, CA, USA), 10 mM non-essential amino acids (Life Technologies), 1 mM HEPES (Life Technologies), 20 mM 2-mercaptoethanol, 100 U/ml penicillin, and 100 mg/ml streptomycin (Life Technologies). Melanoma cell lines were either left untreated over 72 h or treated with recombinant IFN-γ (1000 U/ml IFN-γ, PeproTech, Rocky Hill, NJ, USA).

### mRNA expression analysis

#### TCGA cohort

The mRNA expression data was generated using the Illumina HiSeq 2000 RNA Sequencing Version 2 analysis (Illumina, Inc., San Diego, CA, USA). Expression data of level 3 were provided by the TCGA Research Network. Data were available from *N* = 468 patient samples. Normalized counts (n.c.) per genes were calculated using the SeqWare framework *via* the RSEM (RNA-Seq by Expectation Maximization) algorithm [47].

#### Patient cohort provided by Liu et al.

Liu and colleagues performed the extraction of mRNA from *N* = 121 FFPE melanoma tissue obtained before anti-PD-1 therapy using the QIAGEN AllPrep DNA/RNA Mini Kit (Qiagen, Hilden, Germany). RNA sequencing was performed using the Illumina sequencing platform and TPM levels were reported [25].

#### Melanoma cell lines

Whole-transcript expression data generated with the Human Exon 1.0 ST Array (Affymetrix, Inc., Santa Clara, CA, USA) was obtained from *N* = 19 melanoma cell lines (*N* = 12 lymph node metastases, *N* = 7 brain metastases; GSE44662) [32]. We used mean values of the probe sets 3161114, 3161115, 3161120, 3161126, 3161135, 3161136, 3161137, 3161140, and 3161142 (Fig. 1).

### Methylation analysis

Data on gene methylation (*β*-values) were obtained from the TCGA Research Network and downloaded from the UCSC Xena browser (https://www.xena.ucsc.edu). Data were available from *N* = 470 patient samples. In addition, *β*-values of melanoma cell lines were downloaded from Marzese et al. (GSE44662) [32]. Both methylation studies were performed using the Infinium HumanMethylation450 BeadChip (Illumina, Inc.).

We performed qMSP analysis of additional melanoma cell lines and FFPE melanoma tissues using bisulfite-converted DNA prepared by means of the innuCONVERT Bisulfite All-In-One Kit (Analytik Jena, Jena, Germany) according to the manufacturer’s instructions. Prior to bisulfite conversion, we macrodissected tumor areals from FFPE melanoma tissue sections mounted on glass slides. Our *PD-L2* qMSP assay was duplexed with an *ACTB* assay in order to quantify the total amount of DNA. We used a 100% methylated calibrator sample (CpGenome^TM^ Universal Methylated DNA, Millipore, MA, USA) for the calibration of the qMSP results. qMSP reactions were performed in 20 μl volumes containing 20 ng bisulfite-converted sample and calibrator DNA (quantified via UV–VIS spectrophotometry). We performed triplicate measurements of each sample and the calibrator. PCR buffer conditions were used as previously described [48]. Real-time PCR was carried out using a 7900HT Fast Real-Time PCR system (Applied Biosystems, Waltham, MA, USA) applying the following temperature profile: 20 min at 95 °C and 40 cycles with 2 s at 62 °C, 60 s at 56 °C (each at 100% ramp rate), and 15 s at 95 °C (at 75% ramp rate). Percentage methylation levels were calculated using cycle threshold (CT) values according to the ΔΔCT method as described before [48]. The following oligonucleotides and final concentrations per PCR reaction were used: *ACTB* probe: ATTO 647N-accaccacccaacacacaataacaaacaca-BHQ-2 (0.125 μM); *ACTB* forward primer: gtgatggaggaggtttagtaagtt (0.125 μM); *ACTB* reverse primer: ccaataaaacctactcctcccttaa (0.125 μM); *PD-L2* probe: 6-FAM-ttatttttatgttacggtaaattttaa-BHQ-1 (0.4 μM); *PD-L2* forward primer: aaaattttttaaataagttaggttttc (0.3 μM); and *PD-L2* reverse primer: caaaaaaacactcaaaatttaacgt (0.3 μM).

### Flow cytometry 

Melanoma cells were stained with the following antibodies according to standard procedures: fluorochrome-conjugated monoclonal antibody specific for human PD-L2 (Clone 24F.10C12, BioLegend, San Diego, CA, USA). Apoptosis induction and cell cycle arrest were analyzed using FITC Annexin V Apoptosis Detection Kit (BioLegend). Data were acquired with a FACSCanto flow cytometer (BD Biosciences, San Jose, CA, USA) and analyzed with FlowJo software (V7.6.5 for Windows, TreeStar, Ashland, OR, USA).

### Statistics

Statistical analyses were performed using SPSS, version 23.0 (SPSS Inc., Chicago, IL, USA). Analyses regarding potential correlations of characteristics were calculated using Spearman’s rank correlation (Spearman’s *ρ*). Mean value comparisons were performed using Wilcoxon–Mann–Whitney *U* (two groups) and Kruskal–Wallis (> 2 groups) test. One-way ANOVA and post-hoc Bonferroni test were applied to perform multiple comparisons between groups. In order to reduce the influence of age-related deaths, survival was censored after 5 years (1825 days). Kaplan–Meier method and Cox proportional hazards regression were used for the performance of survival analyses. Progression-free survival (PFS) was defined as the time between the first application of anti-PD-1 antibody and the date of documented disease progression. Overall survival (OS) was defined as time between initial diagnosis and death or last contact, respectively. For Kaplan–Meier analysis methylation levels and mRNA expression levels were dichotomized based on an optimized cutoff (lowest *P* value). Cox proportional hazards analyses were performed with log2-transformed methylation and mRNA expression data (mRNA expression levels of 0 n.c. were set to 0.1 prior to log2-transformation). *P* values refer to log-rank and Wald tests. Two-sided *P* values less than 0.05 were considered statistically significant.

## Data Availability

The datasets generated during and/or analyzed during the current study are available from the corresponding author on reasonable request.

## Abbreviations

95% CI: 95% Confidence interval
B2M: Beta-2 microglobulin
CpG: 5'-cytosine-phosphate-guanosine-3'
CR: Complete response
CTLA4: Cytotoxic T-lymphocyte–associated protein 4
FFPE: Formalin-fixed and paraffin-embedded
HR: Hazard ratio
IHC: Immunohistochemistry
IFN-γ, IFNG: Interferon gamma
JAK: Janus kinase
mRNA: Messenger ribonucleic acid
NA: Not applicable
NK cells: Natural killer cells
OS: Overall survival
PCR: Polymerase chain reaction
PD: Progressive disease
PD-1: Programmed cell death 1
PD-L1: Programmed cell death 1 ligand 1
PD-L2: Programmed cell death 1 ligand 2
PFS: Progression-free survival
PR: Partial response
qMSP: Quantitative methylation-specific PCR
RNA-Seq: RNA sequencing
SD: Stable disease
STAT: Signal transducer and activator of transcription
TCGA: The Cancer Genome Atlas
TILs: Tumor-infiltrating lymphocytes
TPM: Transcripts per million
